# Supplementary material for: Inflammatory signals from fatty bone marrow support DNMT3A driven clonal hematopoiesis
Source: Nat Commun. 2023 Apr 12;14:2070. doi: 10.1038/s41467-023-36906-1 (PMC10097668; doi:10.1038/s41467-023-36906-1)
Supplement: Supplementary file 1 — Supplementary Information [file 41467_2023_36906_MOESM1_ESM.pdf]

Supplementary information

Inflammatory signals from fatty bone marrow support *DNMT3a* driven clonal hematopoiesis

Zioni N[1],Bercovich A[2], Chapal-Ilani N[1], Tal Bacharach[1], Rappoport N[2,3], Solomon A[4], Avraham R[4], Kopitman E[5], Porat Z[5], Sacma M[6], Hartmut G[6], Scheller M[7], Müller-Tidow C[8,9,10], Lipka D[10], Shlush E[11], Minden M[12,13,14,15], Kaushansky N*[1], and Shlush LI.*[#1,16]

[1]Department of Molecular Cell Biology, Weizmann Institute of Science, Rehovot, Israel; [2]Department of Computer Science and Applied Mathematics, Weizmann Institute of Science, Rehovot, Israel;

[3]Blavatnik School of Computer Science, Tel Aviv University, Tel Aviv, Israel

[4]Departemnt of Biological Regulation, Weizmann Institute of Science, Rehovot, Israel

[5]Life sciences Core Facilities, Weizmann Institute of Science, Rehovot, Israel;

[6]Institute of Molecular Medicine Ulm University, Ulm, Germany;

[7]Department of Medicine, Hematology, Oncology and Rheumatology, University Hospital Heidelberg, Heidelberg, Germany;

[8]Department of Internal Medicine V, Heidelberg University Hospital, Heidelberg, Germany;

[9]European Molecular Biology Laboratory (EMBL), Heidelberg, Germany;

[10]German Cancer Consortium (DKTK) and German Cancer Research Center (DKFZ), Partner Site Heidelberg, Heidelberg, Germany;

[11]Ivf unit, Galilee Medical Center, Nahariya, Israel;

[12]Princess Margaret Cancer Centre, University Health Network (UHN), Toronto, ON Canada;

[13]Department of Medical Biophysics, University of Toronto, Toronto, ON Canada;

[14]Department of Medicine, University of Toronto, Toronto, ON Canada;

[15]Division of Medical Oncology and Hematology, University Health Network, Toronto, ON Canada and Hematology, University Health Network, Toronto, ON Canada;

[16]Hematology and Bone Marrow Transplantation Institute Rambam Healthcare campus Haifa Israel;

# To whom correspondence should be addressed.

*Theses authors contributed equally

Content: Supplementary figures 1-15

Supplementary Tables 1-4

Extended data

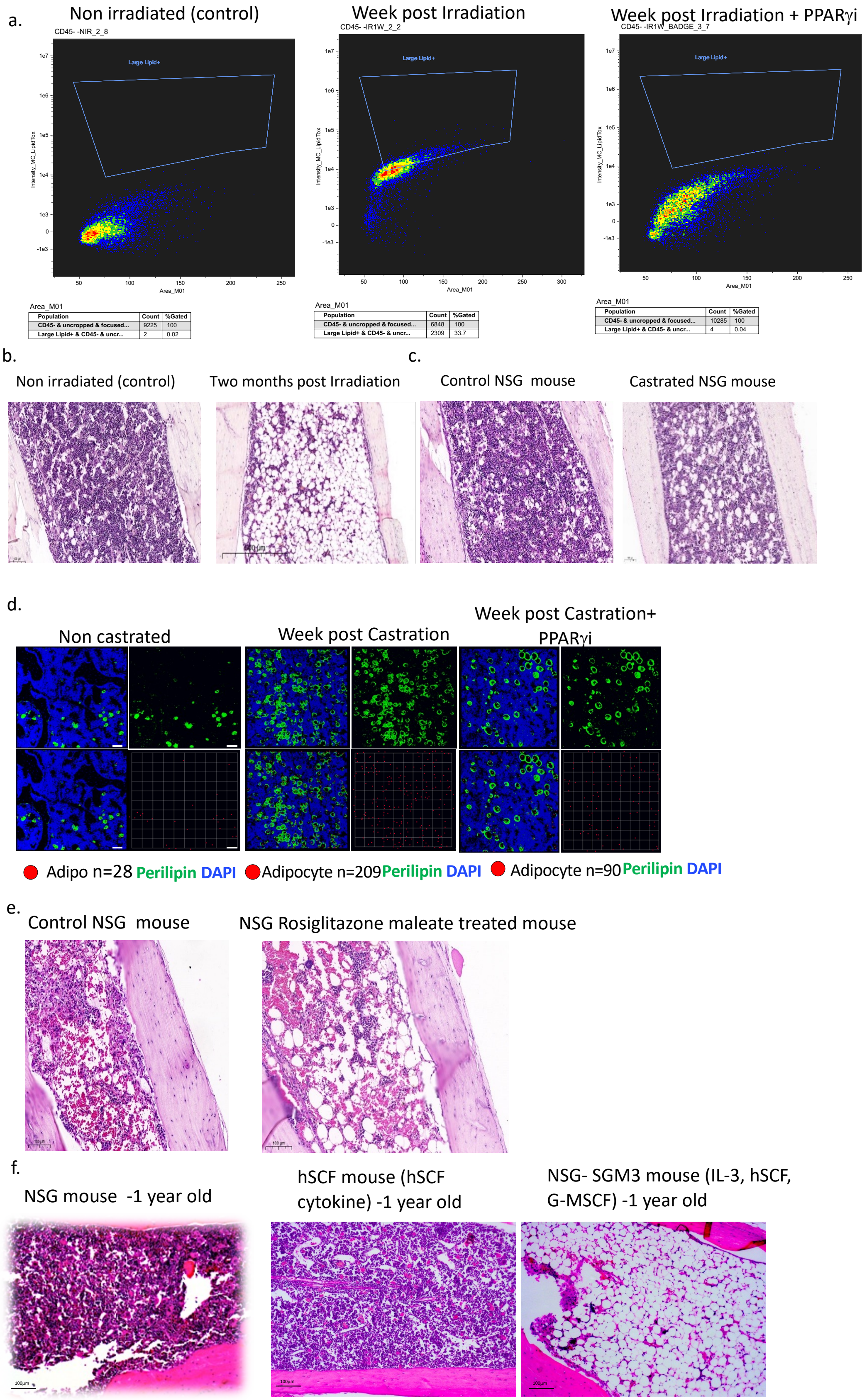

Supplementary Fig 1: FBM establishment in NSG mice. **a**. Image stream analysis of NSG NBM, FBM and FBM + PPARgi mice tibia/femur.CD45 negative cells were stained with LipidTOX™ and DAPI and quantified by image stream. **b.** H&E staining of BM tibia derived from non-Irradiated control normal bone marrow (NBM) (n=5) scale bar: 100μM, and BM derived from two months old NSG mice following irradiation (n=5) scale bar: 500μM. **c.** H&E staining of BM tibia derived from NSG castrated (CAS) mouse month following castration. scale bar: 100μM **d.** Representative 3D whole-mount immunofluorescencestaining of epiphyseal-metaphyseal BM long bonesderived from Castrated NSG mice and castrated mice treated with PPARgi. Adipocytes are depicted by Perilipin expression and by distinctive unilocular morphology in the DIC (differentialinterference contrast) channel. DAPI in blue. Adipocytes are additionally marked byreddots.1 unit Scale Bar:70.99μm; n=12-14708μm x 708μm x30-50μm stacked images from 3 miceand 2 bones (femur, tibia)each group. **e**. H&E staining of BM tibia derived from NSG mouse that were treated orally with PPAR-γ activator (rosiglitazone maleate) (20 mg/kg/day) for three weeks (n=3). scale bar: 100μM **f.** H&E staining of BM tibia derived from one-year-old NSG (n=3), NSG-hSCF (NSG mice that express human membrane-bound stem cell factor) (n=3) or NSG-SGM3 mice (expressing human IL3, GM-CSF (CSF2) and SCF (KITLG)) (n=3). Shown are the H&E staining of tibial of one experiment out of five independent experiments. scale bar: 100μM

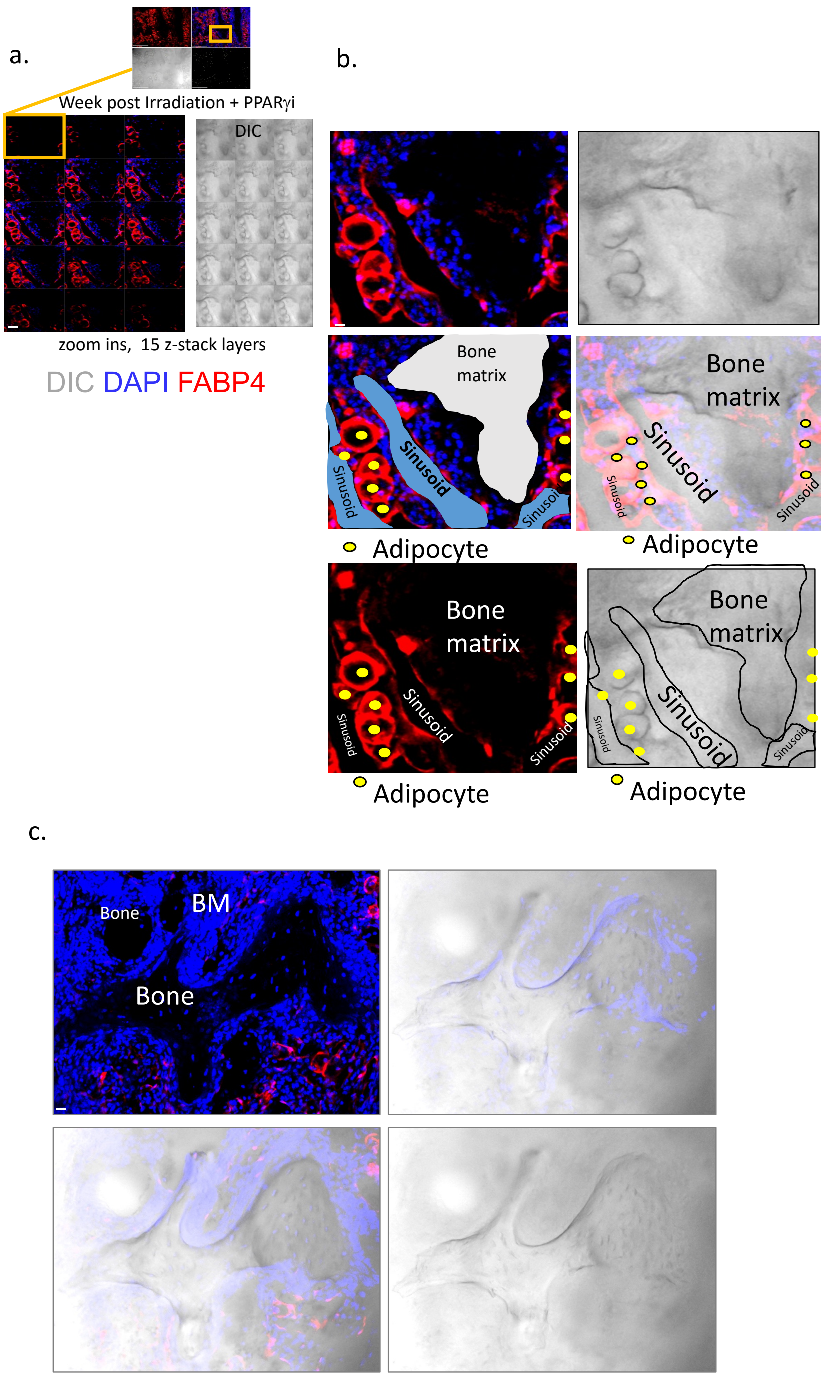

Supplementary Fig 2: Z-stacked confocal images from whole-mounts of bisected NSG mouse bones from fig. 1f. a. Structurer and cellular integrity of Epiphyseal/metaphysical BM regions. b. Detection of typical unilocular morphology of adipocytes was use by DIC (differential interference contrast) channel and also other structures. c. Use of protective whole-mounts, prevent collapsing of sinusoidal vessels, the “empty” spaces here are trabecular bone structures. Data shown is representative of three independent experiments. scale bar: 100μM

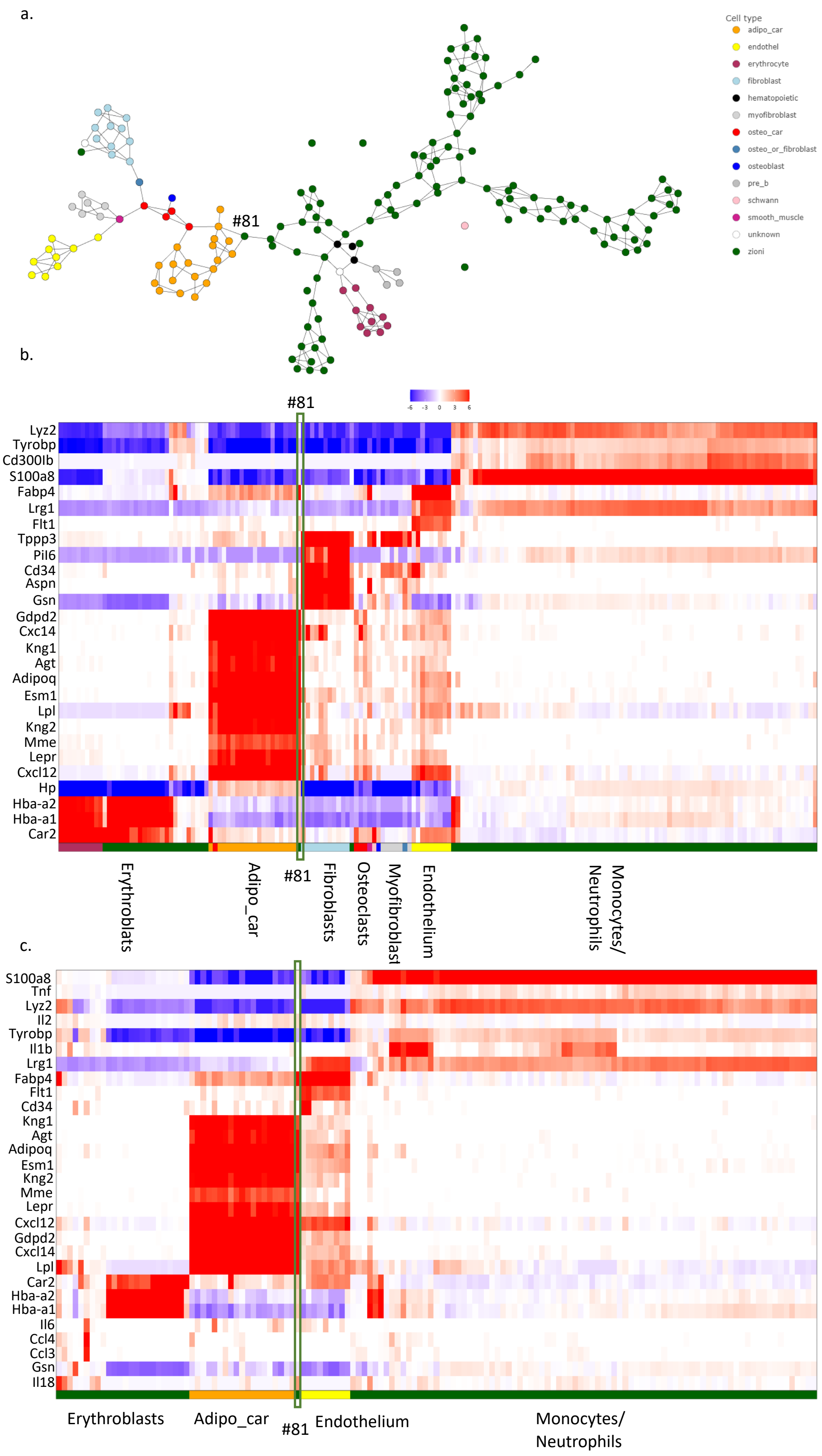
a.
Cell type
adipo_car
endothel
erythrocyte
fibroblast
hematopoietic
myofibroblast
osteo_car
osteo_or_fibroblast
osteoblast
pre_b
schwann
smooth_muscle
unknown
zioni
#81
b.
#81
-6 -3 0 3 6
Lyz2
Tyrobp
Cd300lb
S100a8
Fabp4
Lrg1
Flt1
Tppp3
Pil6
Cd34
Aspn
Gsn
Gdpd2
Cxc14
Kng1
Agt
Adipoq
Esm1
Lpl
Kng2
Mme
Lepr
Cxcl12
Hp
Hba-a2
Hba-a1
Car2
Erythroblats
Adipo_car
#81
Fibroblasts
Osteoclasts
Myofibroblast
Endothelium
Monocytes/
Neutrophils
c.
S100a8
Tnf
Lyz2
Il2
Tyrobp
Il1b
Lrg1
Fabp4
Flt1
Cd34
Kng1
Agt
Adipoq
Esm1
Kng2
Mme
Lepr
Cxcl12
Gdpd2
Cxcl14
Lpl
Car2
Hba-a2
Hba-a1
Il6
Ccl4
Ccl3
Gsn
Il18
Erythroblasts
Adipo_car
#81
Endothelium
Monocytes/
Neutrophils

Supplementary Fig. 3: single nuclei RNA sequencing on BM derived cells. a. BM cells were enriched for adipocytes from both irradiated mice (FBM n=4058 single nuclei) (n=3) and the BADGE control mice (n=3428 single nuclei) (n=3). Single nuclei were isolated and analyzed by 10X genomics. The Metacell platform[1,2] was used to produce the UMAP which was also integrated with single cell RNAseq data from Baccin et.al[3]. Cell type annotations were performed by the same genes as in Baccin et.al. Cells were divided into 181 Metacells. Metacell #81 (circled) was enriched in cells from our cohort (Zioni dark green) and was clustered together with cells designated as Cxcl12-abundant-reticular (CAR) Adipocytes (Adipo_car orange). The UMAP and other features of the integrated model can be observed in the following link: (https://tanaylab.weizmann.ac.il/MCV/FBM/Single_nuclei_for_adipocytes/). **b**. A heatmap of key gene markers defining mouse mesenchymal cells of the integrated data by Baccin et.al. (Erythroblats-purple; Adipo-CAR-orange; Fibroblasts-light blue; Endothelium-yellow; Myofibroblasts-grey; Osteoblats-red) and cells of the current cohort (Zioni in dark green). Most of the Zioni et.al cells were assigned to a neutrophil/monocyte cells, while few were similar to erythroblasts. One Metacells#81 was clustered together with Adipo-car cells. **c**. A heatmap of key gene markers defining mouse mesenchymal cells of the integrated data by Baccin et.al. (Adipo-CAR-orange; Endothelium-yellow;) and cells of the current cohort (Zioni in dark green), which we assigned to either Erythroblasts or monocyte/neutrophil based on gene expression.

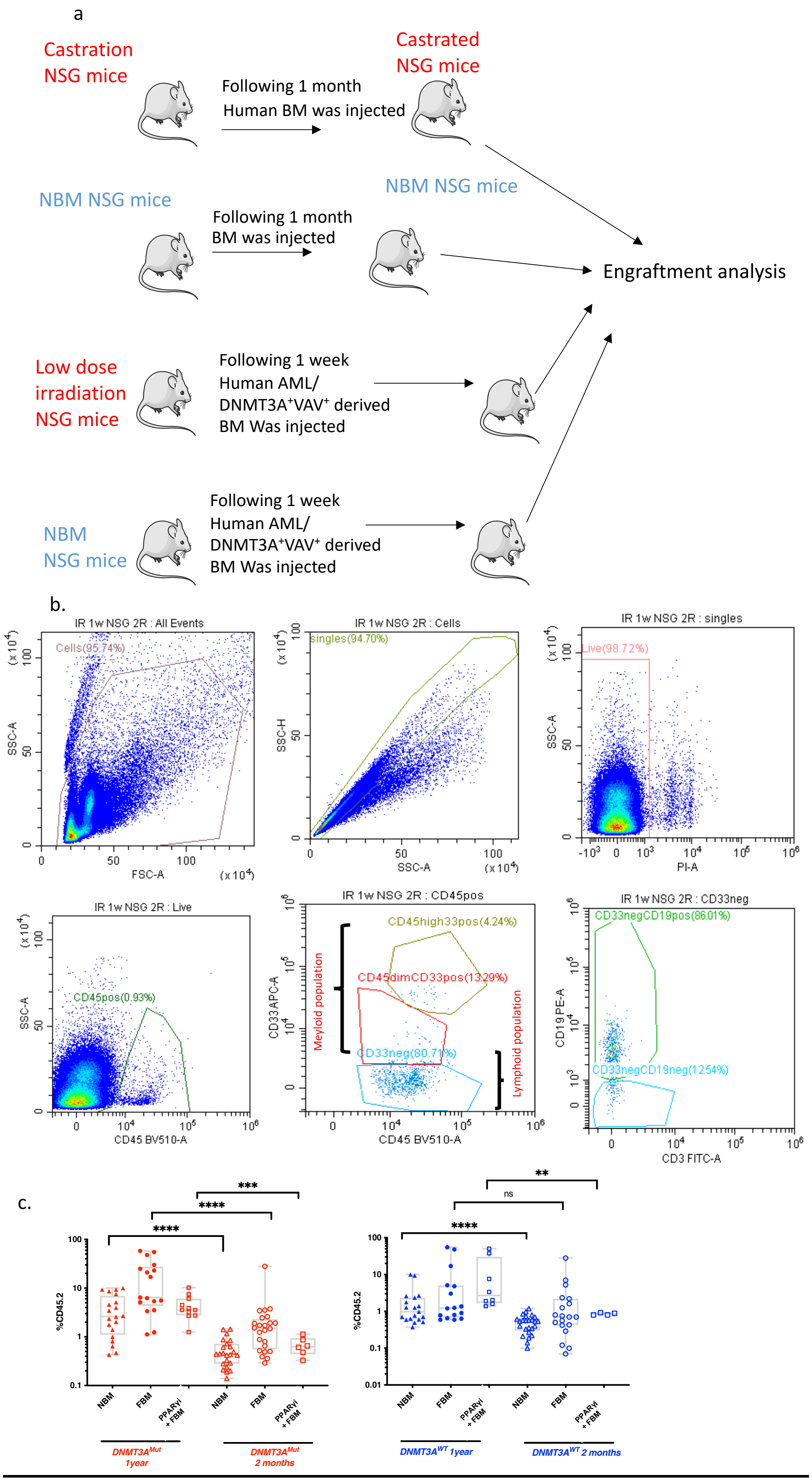
a
Castration NSG mice
Following 1 month Human BM was injected
Castrated NSG mice
NBM NSG mice
Following 1 month BM was injected
NBM NSG mice
Engraftment analysis
Low dose irradiation NSG mice
Following 1 week Human AML/ DNMT3A+VAV+ derived BM Was injected
NBM NSG mice
Following 1 week Human AML/ DNMT3A+VAV+ derived BM Was injected
b.
IR 1w NSG 2R : All Events
Cells(95.74%)
SSC-A
FSC-A
IR 1w NSG 2R : Cells
singles(94.70%)
SSC-H
SSC-A
IR 1w NSG 2R : singles
Live(98.72%)
SSC-A
PI-A
IR 1w NSG 2R : Live
CD45pos(0.93%)
SSC-A
CD45 BV510-A
IR 1w NSG 2R : CD45pos
CD45high33pos(4.24%)
CD45dimCD33pos(13.29%)
CD33neg(80.71%)
Meyloid population
Lymphoid population
CD33 APC-A
CD45 BV510-A
IR 1w NSG 2R : CD33neg
CD33negCD19pos(86.01%)
CD33negCD19neg(12.54%)
CD19 PE-A
CD3 FITC-A
c.
***
****
****
%CD45.2
NBM
FBM
PPARγi + FBM
DNMT3A^Mut 1year
DNMT3A^Mut 2 months
**
ns
****
DNMT3A^WT 1year
DNMT3A^WT 2 months

<u>Fig. 4</u>: a. Schematic presentation of different models used in this study. b. Multilineage engraftment of AML patient-derived. FACs analysis CD3 depleted 1x10^6 AML primary human cells (sample #160005) (50% *hDNMT3A*^{R882H}, 50% *NPM1c)*. Eight weeks later mice were sacrificed, and BM was flashed from tibias and femurs. A multi-lineage engraftment is defined when a subpopulation of B cell progenitors (CD33- CD3- cells expressing CD19+) can be identified. **c.** *DNMT3A*^{Mut} cells derived from one-year-old mice injected into FBM had the most significant growth advantage with a tenfold increase in comparison to NBM and PPARgi controls. Data presented as box and whiskers min to max. All comparisons were performed using a two-tailed, non-paired, nonparametric Mann-Whitney test compare ranks. ***p<0.005, ***p<0.0005, ****p<0.00005, n.s – not significant. *DNMT3A*^{Mut} 1year vs. *DNMT3A*^{Mut} 2months: NBM vs. NBM p<0.00001, FBM vs. FBM p=0.00001, FBM+ PPARγi vs. FBM+ PPARγi p=0.0002. *DNMT3A*^{WT} 1year vs. *DNMT3A*^{WT} 2months: NBM vs. NBM p<0.00001, NBM vs. NBM p=n.s, FBM+ PPARγi vs. FBM+ PPARgi p=0.004. *DNMT3A*^{Mut} 1 year: NBM (n=20), FBM (n=17), FBM+ PPARγI (n=11). *DNMT3A*^{Mut} 2 months: NBM (n=23), FBM (n=24), FBM+ PPARγI (n=6). *DNMT3A*^{WT} 1 year: NBM (n=13), FBM (n=12), FBM+ PPARγI (n=8). *DNMT3A*^{WT} 2 months: NBM (n=24), FBM (n=17), FBM+ PPARγI (n=4). Source data are provided as a Source Data file. Mice image was Created with https://smart.servier.com.Mice figures were drawn by using pictures from Servier Medical Art. Servier Medical Art by Servier is licensed under a Creative Commons Attribution 3.0 Unported License (https://creativecommons.org/licenses/by/3.0/).

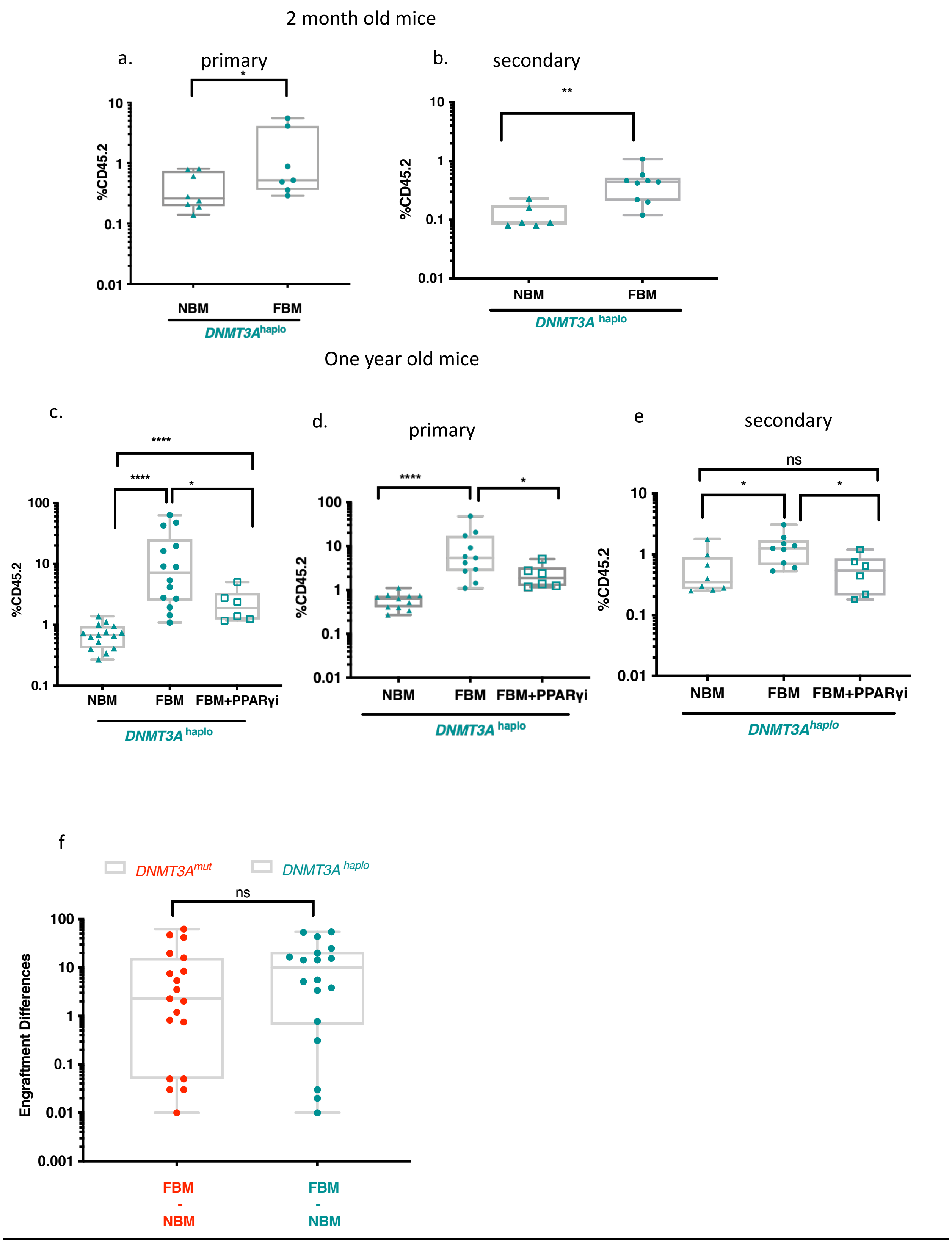

2 month old mice
a.
primary
*
%CD45.2
10
1
0.1
0.01
NBM
FBM
DNMT3A haplo
b.
secondary
**
%CD45.2
10
1
0.1
0.01
NBM
FBM
DNMT3A haplo
One year old mice
c.
****
****
*
%CD45.2
100
10
1
0.1
NBM
FBM
FBM+PPARγi
DNMT3A haplo
d.
primary
****
*
%CD45.2
100
10
1
0.1
0.01
NBM
FBM
FBM+PPARγi
DNMT3A haplo
e
secondary
ns
*
*
%CD45.2
10
1
0.1
0.01
NBM
FBM
FBM+PPARγi
DNMT3A haplo
f
DNMT3A mut
DNMT3A haplo
ns
Engraftment Differences
100
10
1
0.1
0.01
0.001
FBM - NBM
FBM - NBM

Supplementary Fig. 5: Engraftment of *DNMT3A*^{haplo} derived BM cells in FBM NSG mice. a. FACs analysis of young-two-month-old 6x10^6 *DNMT3A*^{haplo} (CD45.2) BM derived cells transplanted to normal bone marrow (NBM) (n=8) and fatty bone marrow (FBM) (NSG mice are CD45.1) (n=7). Eight weeks cells following transplantation, BM was flashed from tibia/femur and expression of mCD45.2 was measured by FACs. Engraftment was assessed according to presence of ≥0.1% mCD45.2 cells. **b**. Self-renewal of *DNMT3A*^{haplo} derived BM cells in FBM NSG mice. Primary transplantation of *DNMT3A*^{haplo} was performed as detailed in a. Then, a secondary transplantation was performed to FBM NSG mice (n=6, n=7 respectively). NBM vs. FBM p=0.0047. **c.** FACs analysis of one-year-old *DNMT3A*^{haplo}*,* 6x10^6 BM derived cells transplanted to control (n=15), to a week following Irradiation (n=14) and to Irradiated NSG mice (CD45.1) treated with PPARgi (n=6) performed as detailed in a. NBM vs. FBM p<0.00001, FBM vs. FBM+ PPARgi p= 0.0326. NBM vs. FBM+ PPARgi p<0.00001**. d.** Primary transplantation of one-year *DNMT3A*^{haplo} BM derived cells to NBM mice (n=11), and to FBM mice (n=11) and to Irradiated NSG mice treated with PPARgi (FBM+ PPARgi) (n=6). NBM vs. FBM p<0.00001, FBM vs. FBM+ PPARgI p=0.0477 **e**. Secondary transplantation of cells from d. to FBM NSG mice (n=8, n=9, n=6 respectively). NBM vs. FBM p=0.0206, FBM vs. FBM+ PPARgI p=0.0128. **f**. Differences (FBM-NBM) between engraftment of middle-aged *DNMT3A*$^{Mut-}$ and *DNMT3A*^{haplo} BM derived cells when transplanted to FBM. Each dot represents a mouse. (*DNMT3A*^{Mut} n=19, *DNMT3A*^{haplo} n=18). Data in this figure presented as box and whiskers min to max . All comparisons were performed using a two-tailed, non-paired, nonparametric Mann-Whitney test compare ranks. ***p<0.005, ***p<0.0005, ****p<0.00005, n.s – not significantSource data are provided as a Source Data file.

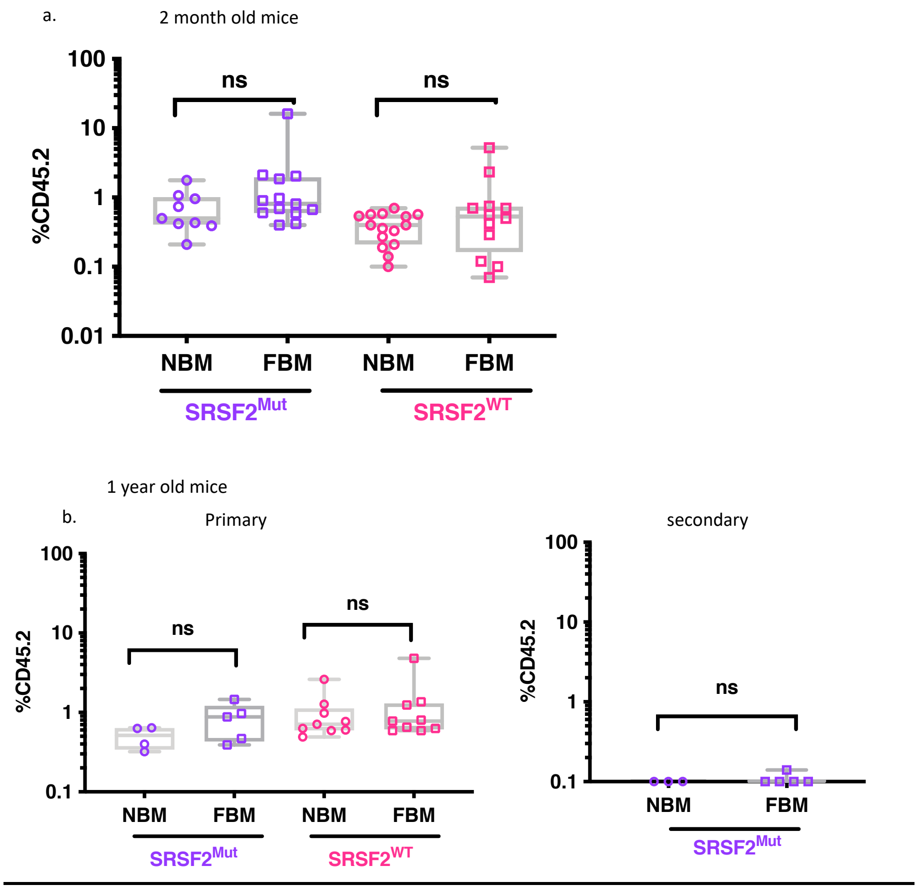

Supplementary Fig. 6: Engraftment analysis of *SRSF2*^{Mut} or control *SRSF2*^{WT} BM derived cells in FBM. a. FACs analysis of young-two-month-old 6x10^^{6} *SRSF2*^{Mut} (purple) or control *SRSF2*^{WT} (pink) (CD45.2) BM derived cells transplanted to normal bone marrow mice (NBM) (*SRSF2*^{WT} to n=15 NSG mice, *SRSF2*^{Mut} to n=9 NSG mice) and to fatty bone marrow (FBM) NSG mice (CD45.1) (*SRSF2*^{WT} to n=12 NSG mice, *SRSF2*^{Mut} to n=13 NSG mice). Eight weeks following transplantation, BM was flashed from tibia/femur and expression of mCD45.2 was measured by FACs. Engraftment was assessed according to presence of ≥0.1% mCD45.2 cells. **b**. Primary transplantation of middle-aged *SRSF2*^{Mut} (purple) (NBM, n= 4; FBM, n=5) or control *SRSF2*^{WT} (pink) (NBM, n=9; FBM, n=9) was performed as detailed in a. Then, a secondary transplantation of middle-aged *SRSF2*^{Mut} (purple) BM derived cells was performed to FBM NSG mice (n=3, n=5 respectively). Data in this figure presented as box and whiskers min to max. n.s – not significant. All comparisons were performed using a two-tailed, non-paired, nonparametric Mann-Whitney test compare ranks. Source data are provided as a Source Data file.

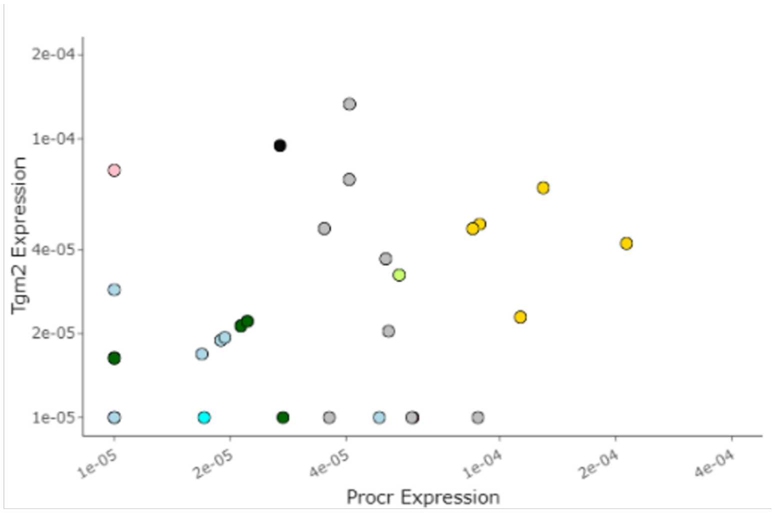

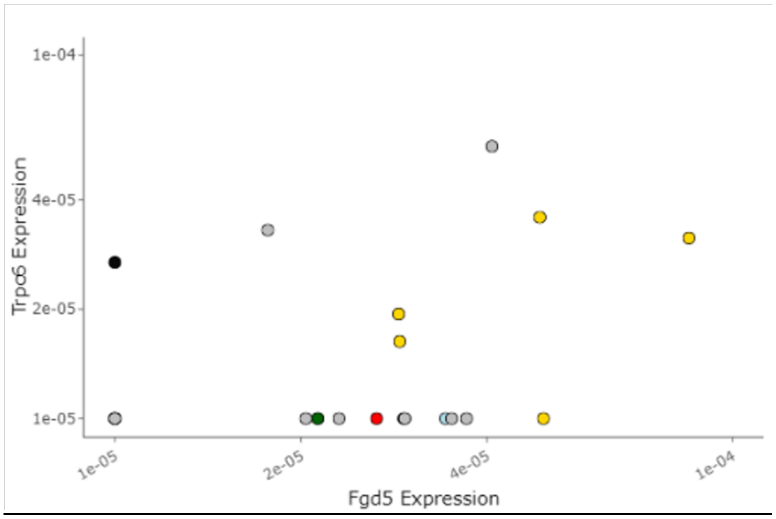

<u>Supplementary Fig. 7:</u> Single cell RNA-seq analysis and cell type designation

Cells from *DNMT3A*^{mut} and *DNMT3A*^{WT} were injected to mice with fatty bone marrow (FBM) and normal bone marrow (NBM). Three days after injection lin-+KIT+ (LK) cells were isolated from mice bone marrow (BM) and underwent single cell RNA-Seq analysis. MetaCell algorithm was used to assign different single cells to Metacells with unique gene programs and cell types 38. Gold, hematopoietic stem cells (HSCs); darkgreen, common myelid progenitors (CMP); lightblue, common lymphoid progenitors, (CLP); cyan, dendritic progenitors (DcP); grey, multipotent progenitors (MPP); darkolivegreen, monocyte progenitors (MonoP); pink, megakaryocyte progenitors (MegK); red, erythroid progenitors (EryP); grey4 (Unknown). Conditions: normal bone marrow (NBM); wild type (wt); fatty bone marrow (FBM); naïve cells- are cells extracted directly from BM of respective mice without transplantation. cre is the cre control. Each signle cell was designated to a Metacell and each Metacell got its cell type designation based on the expression of the main lineage defining HSCs genes. Source data are provided as a Source Data file.

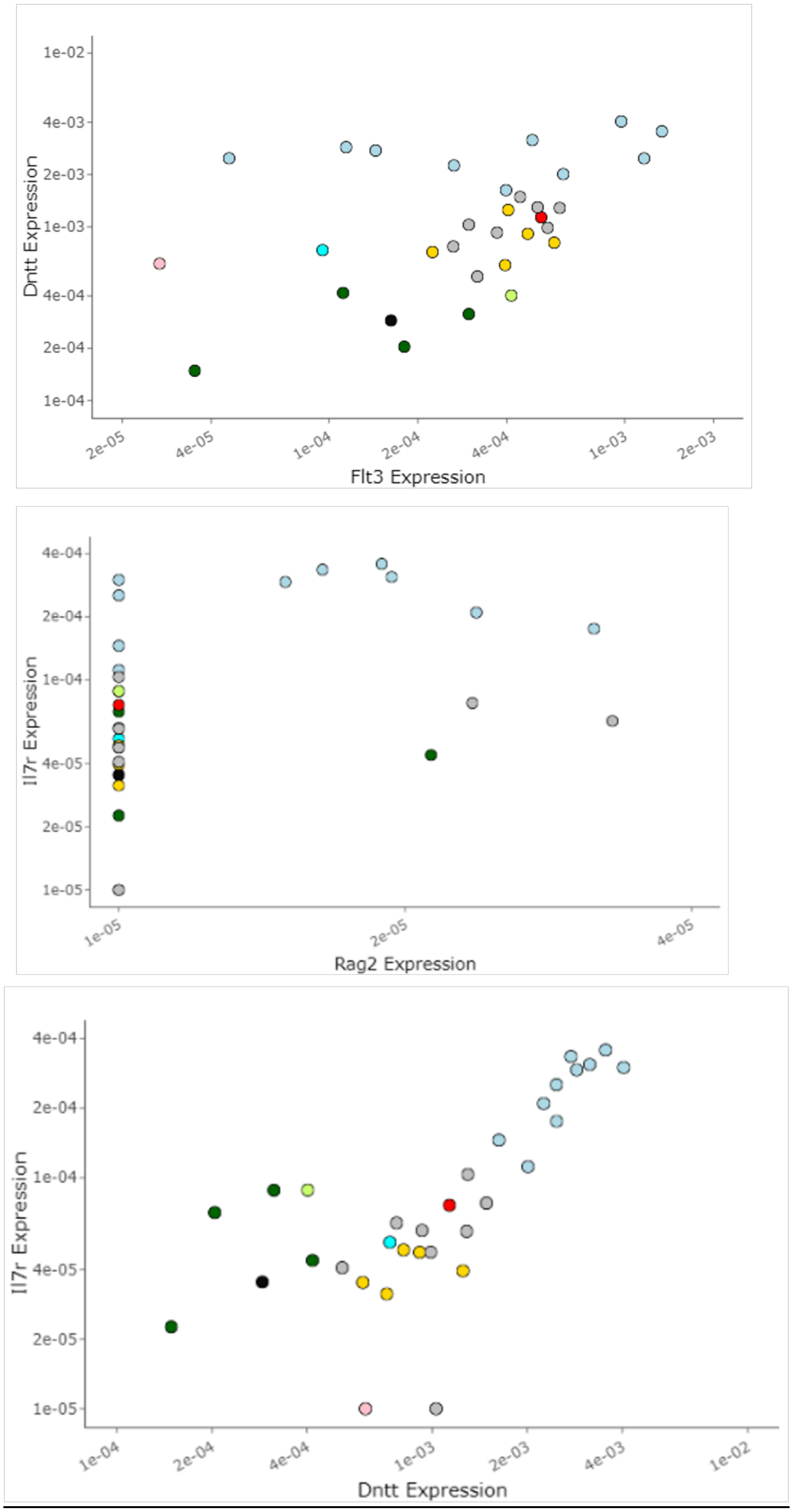

Supplementary Fig. 8 Single cell RNA-seq analysis and cell type designation. As was described in Supplementary Fig 7. Here, each single cell was designated to a Metacell and each Metacell got its cell type designation based on the expression of the main lineage defining CLPs genes. Source data are provided as a Source Data file

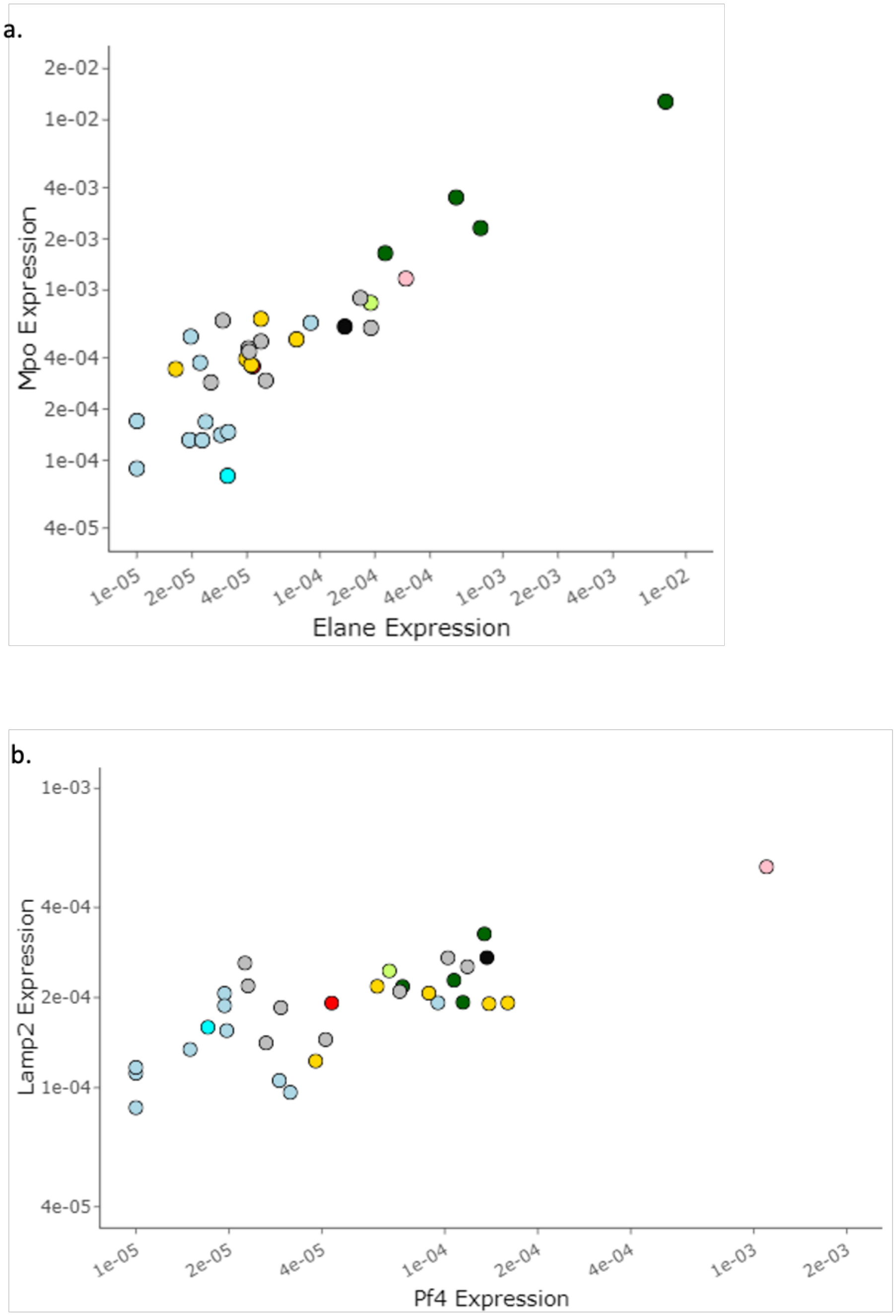

<u>Supplementary Fig. 9</u> Single cell RNA-seq analysis and cell type designation. As was described in Supplementary Fig 7. Here, each single cell was designated to a Metacell and each Metacell got its cell type designation based on the expression of the main lineage defining CMP genes (**a**) and MegK (**b**). Source data are provided as a Source Data file

a.

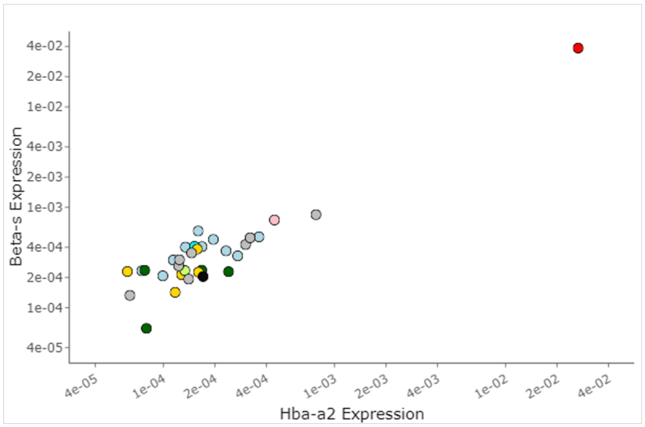

b.

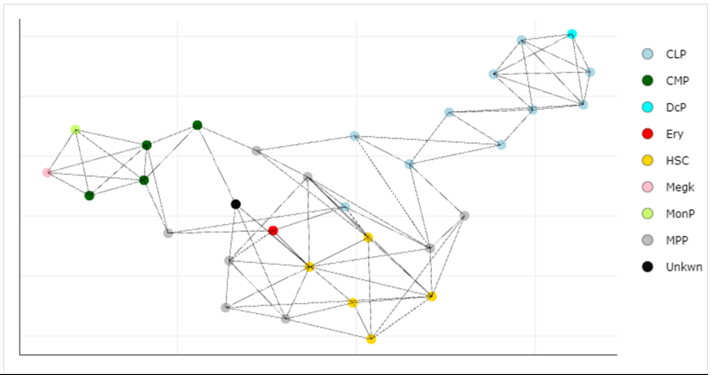

<u>Supplementary Fig. 10:</u> Single cell RNA-seq analysis and cell type designation. As was described in Supplementary Fig 7. Here, each single cell was designated to a Metacell and each Metacell got its cell type designation based on the expression of the main lineage defining Ery genes (**a**) and The Metacell model of the sc-RNA-seq data Supplementary (**b**). Source data are provided as a Source Data file.

a.

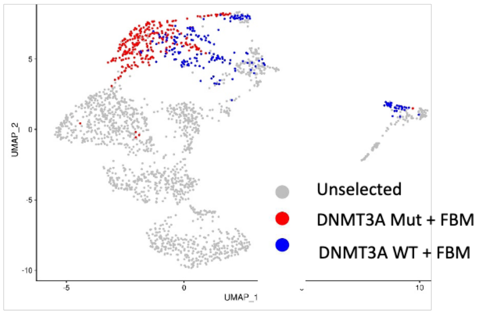

b.

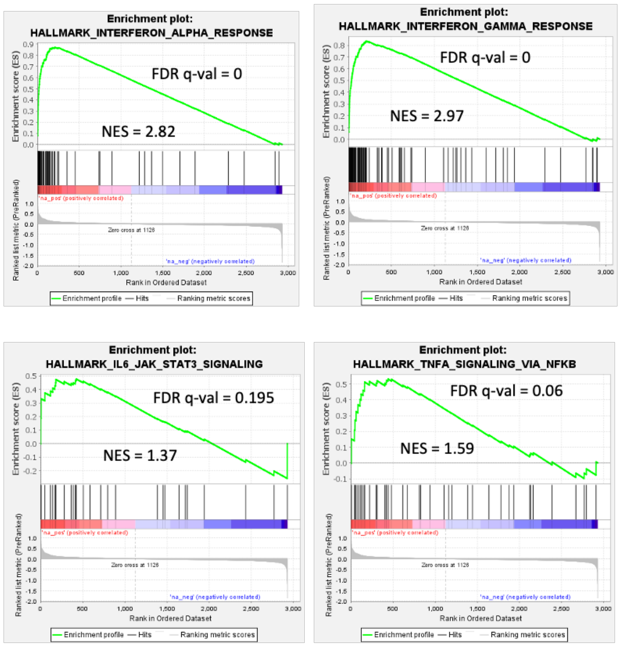

<u>Supplementary Fig. 11:</u> Ranked GSEA analysis of differentially expressed genes between the cluster containing the *DNMT3A*^{Mut} cells exposed to FBM and all other cells.

a. The UMAP clustering of the single cell RNA sequencing data exposed the clustering of *DNMT3A*^{mut} cells exposed to FBM (red cells) almost exclusively to a single cluster (cluster #1 in Supplementary Table 2). *DNMT3A*^{wt} cells exposed to FBM (blue) were separated from the *DNMT3A*^{mut} cells. All other cells which include Mut and Wt cells on normal bone marrow (NBM); naïve cells- are cells extracted directly from BM of respective mice without transplantation and cre control are included in the Unselected group and are marked in grey. **b**. Ranked GSEA analysis on differentially expressed genes between *DNMT3A*^{mut} cells exposed to FBM cluster and other clusters exposed significant enrichment of inflammatory pathways. For each pathway a normalized enrichment score (NES) and false discovery rate (FDR) q value are presented. Source data are provided as a Source Data file.

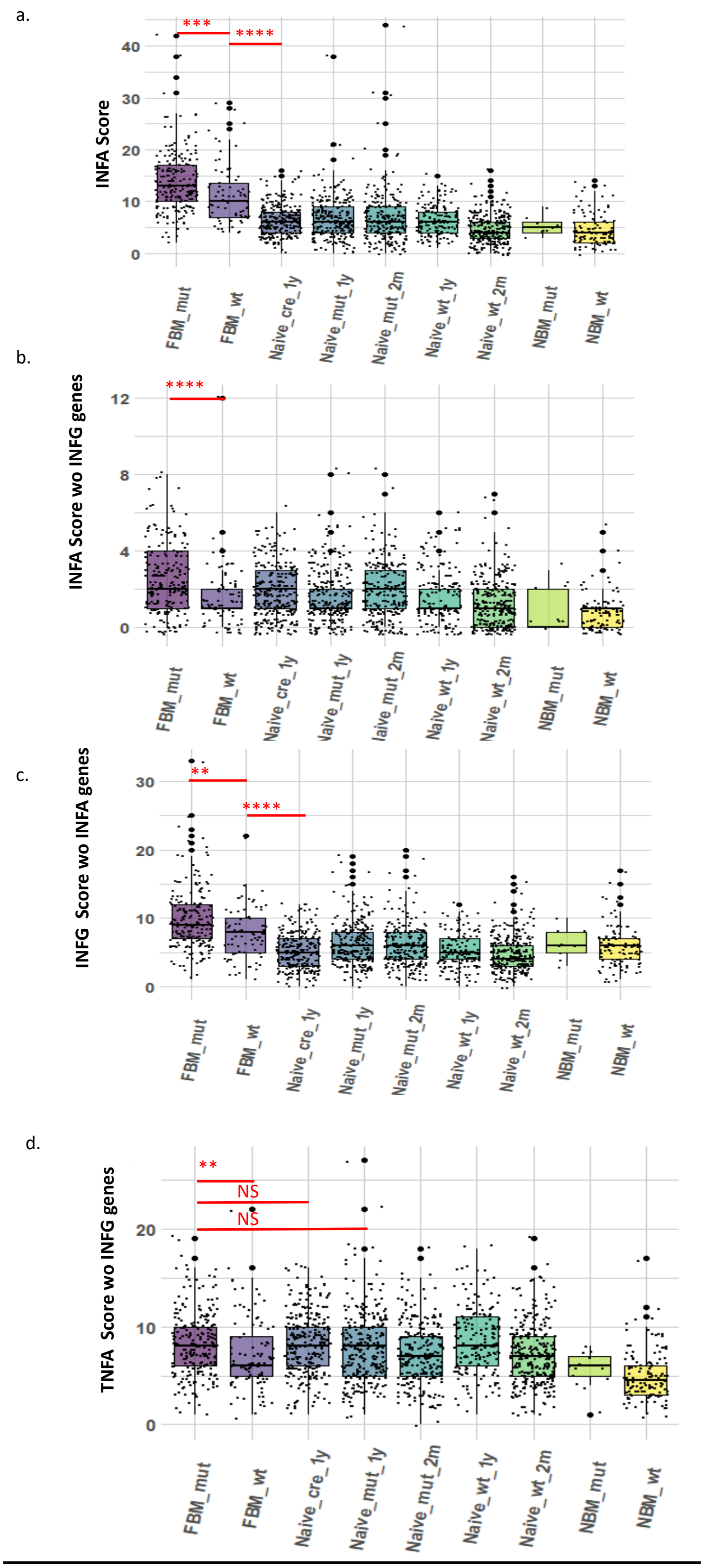

a.
***
****
INFA Score
0
10
20
30
40
FBM_mut
FBM_wt
Naive_cre_1y
Naive_mut_1y
Naive_mut_2m
Naive_wt_1y
Naive_wt_2m
NBM_mut
NBM_wt
b.
****
INFA Score wo INFG genes
0
4
8
12
FBM_mut
FBM_wt
Naive_cre_1y
Naive_mut_1y
Naive_mut_2m
Naive_wt_1y
Naive_wt_2m
NBM_mut
NBM_wt
c.
**
****
INFG Score wo INFA genes
0
10
20
30
FBM_mut
FBM_wt
Naive_cre_1y
Naive_mut_1y
Naive_mut_2m
Naive_wt_1y
Naive_wt_2m
NBM_mut
NBM_wt
d.
**
NS
NS
TNFA Score wo INFG genes
0
10
20
FBM_mut
FBM_wt
Naive_cre_1y
Naive_mut_1y
Naive_mut_2m
Naive_wt_1y
Naive_wt_2m
NBM_mut
NBM_wt

Supplementary Fig. 12: Inflammatory signaling scores in the scRNA-seq data. Ranked GSEA analysis on differentially expressed genes between *DNMT3A*^{mut} cells exposed to FBM cluster and other clusters exposed significant enrichment of inflammatory pathways. An expression score for each single cell was calculated based on the expression of each of the genes in the gene set was calculated. **a.** Interferon alpha (IFNγ) gene set score. FBM_mut vs. FBM_wt p= 0.000114, FBM_wt vs. Naive_cre_1y p= 1.70E-15.
b. INFa gene set without genes shared by the interferon gamma (IFNγ) gene set. FBM_mut vs. FBM_wt p= 1.02E-05. **c.** IFNγ gene set. FBM_mut vs. FBM_wt p= 0.001303, FBM_wt vs. Naive_cre_1y p= 2.89E-09. **d.** Tumor necrosis factor alpha (TNFa) gene set without genes shared with the IFNγ gene set. FBM_mut vs. FBM_wt p= 0.002381. All comparisons were performed using a two-tailed, non-pared, nonparametric Wilcoxon rank sum test with 95% confidence interval with FDR multiple hypothesis. * p<0.05, **p<0.005, ***p<0.0005, ****p<0.00005. Conditions: normal bone marrow (NBM); wild type (wt); fatty bone marrow (FBM); naïve cells- are cells extracted directly from BM of respective mice without transplantation. cre is the cre control.

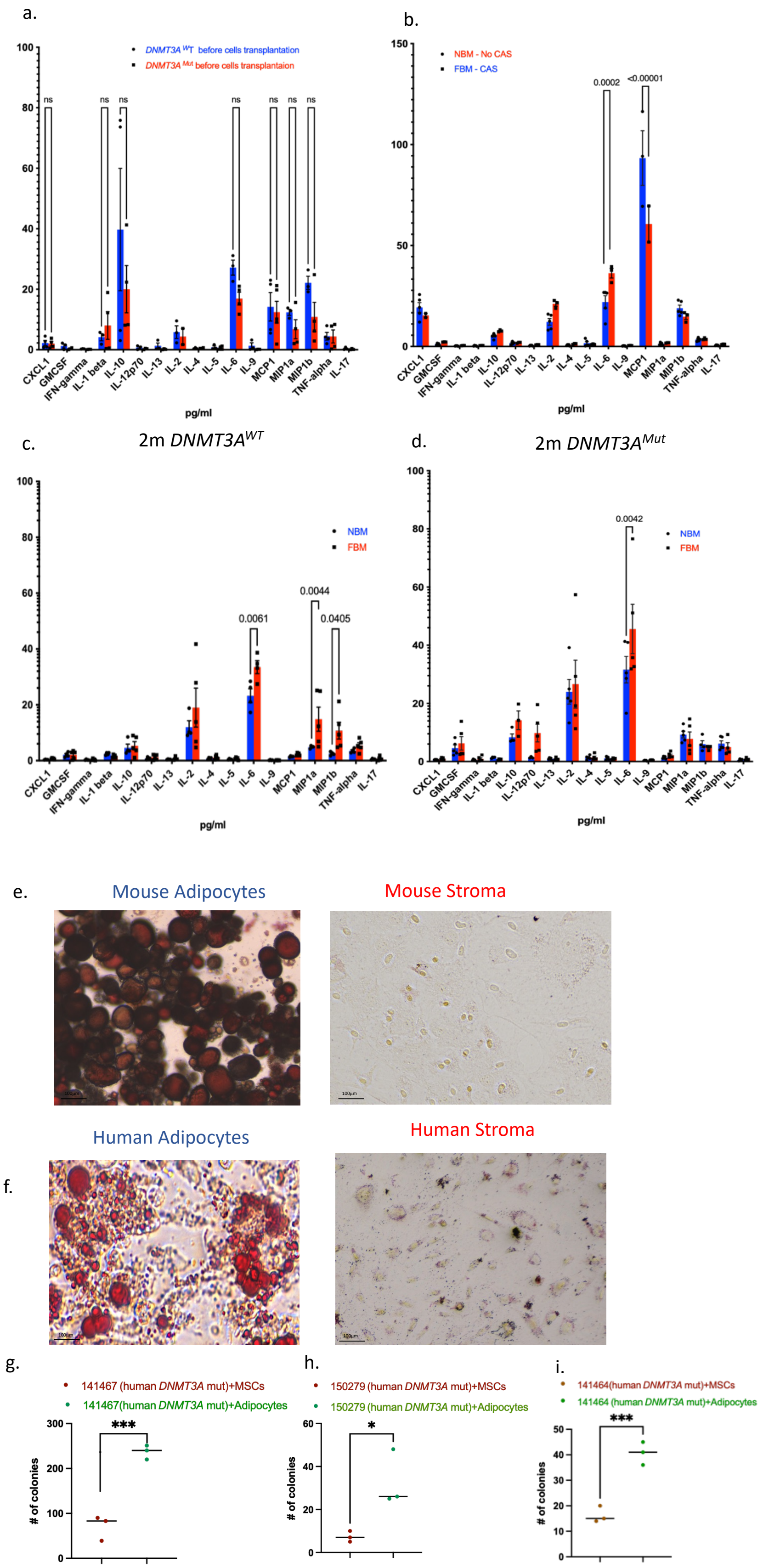

a.
DNMT3A WT before cells transplantation
DNMT3A Mut before cells transplantaion
ns
CXCL1 GMCSF IFN-gamma IL-1 beta IL-10 IL-12p70 IL-13 IL-2 IL-4 IL-5 IL-6 IL-9 MCP1 MIP1a MIP1b TNF-alpha IL-17
pg/ml
b.
NBM - No CAS
FBM - CAS
0.0002
<0.00001
pg/ml
c.
2m DNMT3A WT
NBM
FBM
0.0061
0.0044
0.0405
pg/ml
d.
2m DNMT3A Mut
NBM
FBM
0.0042
pg/ml
e.
Mouse Adipocytes
Mouse Stroma
f.
Human Adipocytes
Human Stroma
g.
141467 (human DNMT3A mut)+MSCs
141467(human DNMT3A mut)+Adipocytes
***
# of colonies
h.
150279 (human DNMT3A mut)+MSCs
150279 (human DNMT3A mut)+Adipocytes
*
# of colonies
i.
141464(human DNMT3A mut)+MSCs
141464 (human DNMT3A mut)+Adipocytes
***
# of colonies

<u>Supplementary Fig. 13:</u> Cytokine analysis of donor *DNMT3A^Mut^* or *DNMT3A^WT^* BM fluid. a. Multiplex cytokines assay of 17 common cytokines analyzed by FACs in middle-aged donor $DNMT3A^{Mut}$ or $DNMT3A^{WT}$ BM fluid. One-year-old donor $DNMT3A^{Mut}$ or $DNMT3A^{WT}$ mice were sacrificed and BM cytokines from tibia/femur were analyzed. Data was analyzed by two-way ANOVA test – Sidaks multiple comparison test. Data are presented as mean values +/- SEM. The figure displays the p value. $DNMT3A^{WT}$ before transplantation n= 4,3,3,3,4,3,3,3,3,3,3,4,3,3,4,3. $DNMT3A^{Mut}$ before transplantation n= 4,3,3,4,4,3,3,3,3,4,4,3,5,4,4,4,3. **b**. FACS based multiplex method of NBM and FBM following castration of NSG mice. Data was analyzed by two-way ANOVA test – Sidaks multiple comparison test. Data are presented as mean values +/- SEM .The figure displays the p value. NBM n=5 for all except for MCP1 n=3. FBM n=3 for all, except for CXCL1 and MCP1 n=2. **c-d.** FACS based multiplex method of NBM and FBM of NSG mice transplanted with 2-months-old $DNMT3A^{Mut}$ or $DNMT3A^{WT}$ BM derived cells. Data was analyzed by two-way ANOVA test – Sidaks multiple comparison test. The figure displays the p value. c. NBM n=4 for all, FBM n=5 except for IL-6 n=4. d. NBM n=5,5,5,4,4,3,5,5,5,5,5,5,5,5,4,5,4. FBM n= 5,5,5,4,3,5,4,5,5,4,5,5,5,5,4,4,4. **e-f** mouse/human MSCs and adipocytes were cultured *in vitro* for ten days. Adipocytes were quantified using oil red staining. Data presented here is representative of three different experiments. scale bar: 100μM. **g-i** Human AML CD34+ cells with a *DNMT3A* mutation (sample #141467, sample #150279, sample #141464) were co cultured with adipocytes/MSCs. After ten days Colony Forming Unit (CFU) assay (n=3 for each sample) were performed, in which cells were cultured in methylcellulose with adipocytes or MSCs-derived media. Data in this figure presented as box and whiskers min to max. All comparisons were performed using a two-tailed, non-paired, Mann-Whitney test compare ranks. * p<0.05, ***p<0.0005. g. 141467 (human $DNMT3A^{Mu}$)+MSCs vs. 141467(human $DNMT3A^{Mu}$)+Adipocytes p=0.0004.h. 150279 (human DNMT3A mut)+MSCs vs. 150279 (human $DNMT3A^{Mu}$)+Adipocytes p=0.0142.i. 141464(human $DNMT3A^{Mut}$)+MSCs vs. 141464 (human $DNMT3A^{Mut}$)+Adipocytes p=0.0008. Source data are provided as a Source Data file.

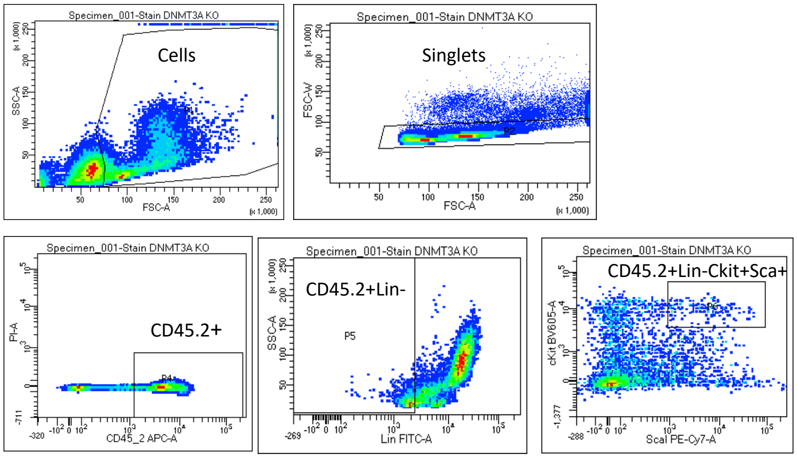

B.

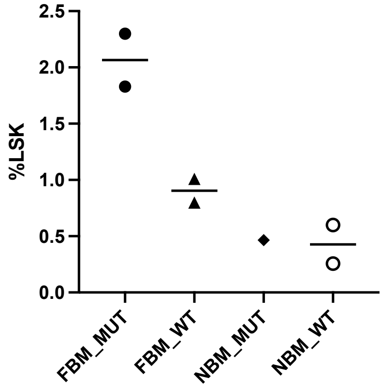

Supplementary Fig. 14: a. Steps of FACs analysis for LSK sorting. Cells were gated by using forward scatter height (FSC-H) vs. Forward scatter area (FSC-A) and FSC-w vs. FSC-A to exclude doublets. To further analyze LSK, cells were gated on CD45.2 cell population from which Lin negative cells were gated**.** Then cKit and Sca1 expression was analyzed on Lin- cells**. b.** Biological replicates of LSK (Data are presented as median) under NBM and FBM.FBM_MUT n=2, FBM_WT n=2, NBM_MUT n=1, NBM_WT n=2. Source data are provided as a Source Data file.

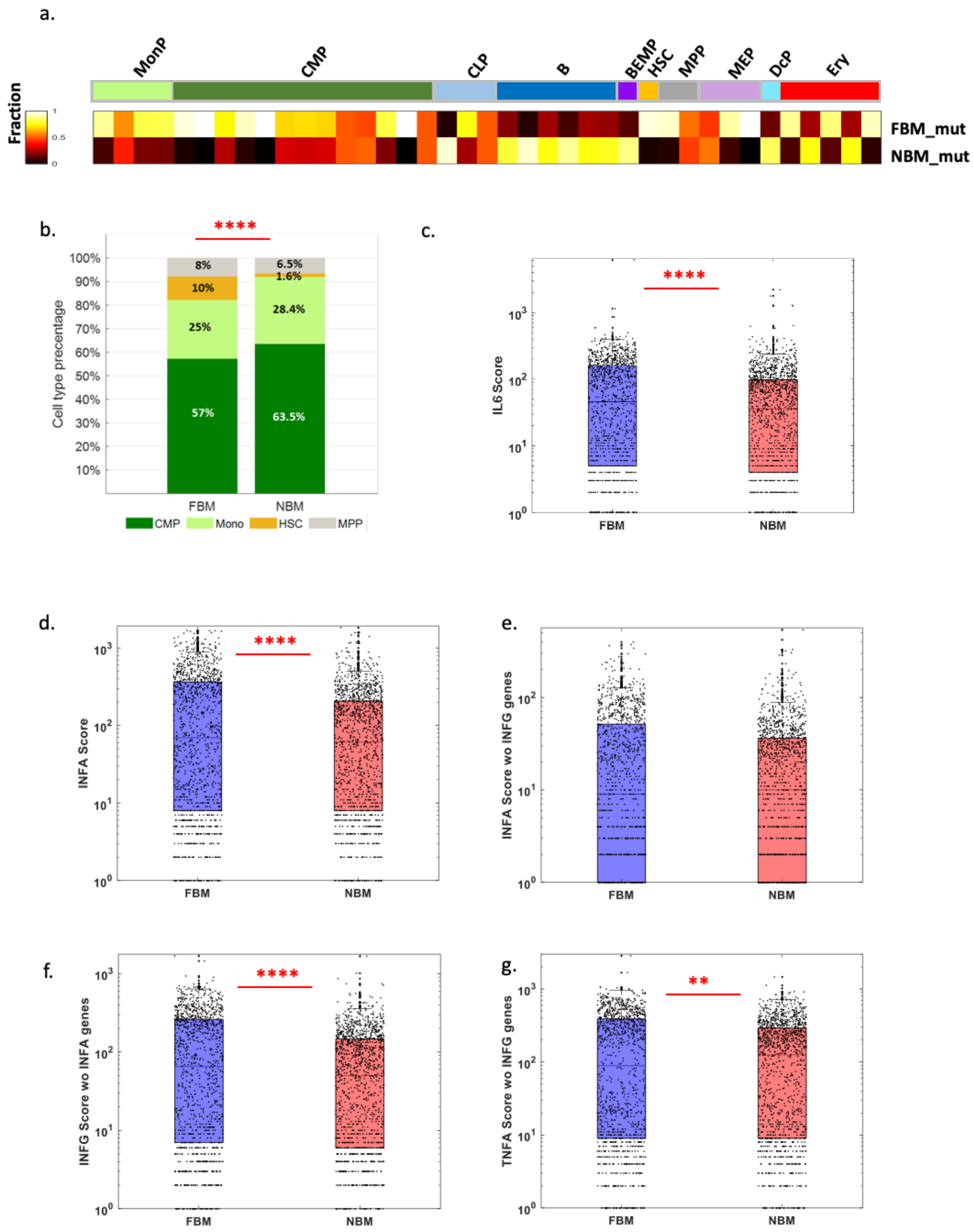

a.
MonP
CMP
CLP
B
BEMP
HSC
MPP
MEP
DcP
Ery
Fraction
1
0.5
0
FBM_mut
NBM_mut
b.
****
100%
90%
80%
70%
60%
50%
40%
30%
20%
10%
Cell type precentage
8%
10%
25%
57%
6.5%
1.6%
28.4%
63.5%
FBM
NBM
CMP
Mono
HSC
MPP
c.
****
IL6 Score
FBM
NBM
d.
****
INFA Score
FBM
NBM
e.
INFA Score wo INFG genes
FBM
NBM
f.
****
INFG Score wo INFA genes
FBM
NBM
g.
**
TNFA Score wo INFG genes
FBM
NBM

Supplementary Fig. 15: validation of the scRNA-seq results by 10X V3 3' analysis.

Bone marrow (BM) cells derived from one year old *DNMT3A*^{mut} were injected to mice with fatty bone marrow (FBM) and normal bone marrow (NBM). Three days after injection lin-Sca1+KIT+ (LSK) cells were isolated from mice BM and underwent single cell RNA-Seq analysis. **a.** MetaCell algorithm was used to assign different single cells to Metacells with unique gene programs and cell types [1]. Gold, hematopoietic stem cells (HSCs); darkgreen, common myeloid progenitors (CMP); lightblue, common lymphoid progenitors, (CLP); cyan, dendritic progenitors (DcP); grey, multipotent progenitors (MPP); darkolivegreen, monocyte progenitors (MonoP); plum megakaryocytic erythroid progenitor (MEP); purple, basophil, eosinophil, mast progenitors (BEMPs); red, erythroid progenitors (Ery); steelblue B cells (B) and . **b.** While cell exposed to FBM demonstrate a marked reduction in HSCs after transplantation *DNMT3A*^{mut} cells exposed to FBM have significantly higher HSCs frequency chi-square ****p<0.0001. **c**. Ranked GSEA analysis on differentially expressed genes between *DNMT3A*^{mut} cells exposed to FBM cluster and other clusters exposed significant enrichment of inflammatory pathways one of them was the IL-6 JAK STAT3 response gene set. An expression score for each single cell was calculated based on the expression of each of the genes in the IL-6 gene set. All comparisons were performed using a two-tailed, non-pared, nonparametric Wilcoxon rank sum test with 95% confidence interval with FDR multiple hypothesis. **p<0.005, *****p<0.00005. NBM vs. FBM P < 10^-8. **d.** Interferon alpha (IFNα) gene set score. NBM vs. FBM P < 10^-7 **e**. INFα gene set without genes shared by the interferon gamma (IFNγ) gene set. NBM vs. FBM P =0.089 **f.** IFNγ gene set. NBM vs. FBM P < 10^-11 **g.** Tumor necrosis factor alpha (TNFα) gene set without genes shared with the IFNγ gene set. NBM vs. FBM P = 0.002. Source data are provided as a Source Data file.

Supplementary Table 1: Distribution of single cells from the different experimental condition across the different Metacells and different HSPCs sub-populations.

Meta_Cell_#	Cell_Type	FBM_cre	FBM_mut	FBM_wt	NBM_cre	NBM_mut	NBM_wt	Naive_cre_1y	Naive_mut_1y	Naive_mut_2m	Naive_wt_1y	Naive_wt_2m			Color	Cell Type
0	gold	0	6	0	0	0	0	44	5	4	30	7			gold	HSCs
1	darkgreen	10	1	9	60	8	7	1	0	0	0	0			darkgreen	CMP
2	lightblue	0	3	0	0	0	0	14	8	16	3	8			lightblue	LP
3	lightblue	0	4	0	0	0	0	16	14	8	6	5			cyan	DcP
4	darkgreen	12	19	19	2	1	4	1	2	0	0	1			grey	MPP
5	cyan	0	1	0	8	0	0	0	10	21	0	12			darkolivegreen1	MonoP
6	grey4	1	20	7	2	1	2	17	2	3	13	2			pink	MegK
7	darkolivegreen1	15	22	6	10	4	4	0	5	5	0	3			red	EryP
8	pink	26	1	1	14	0	3	0	19	12	0	0			grey4	Unknown
9	grey	0	9	0	1	0	0	18	5	11	7	4				
10	red	4	1	0	2	0	1	11	8	8	3	5				
11	gold	0	5	0	1	0	0	23	9	9	12	4				
12	gold	0	16	0	1	0	0	27	4	2	7	9				
13	lightblue	0	6	0	0	0	0	7	17	10	3	26				
14	darkgreen	2	49	1	2	0	0	0	2	0	0	2				
15	darkgreen	9	7	11	20	2	5	0	0	1	2	2				
16	gold	0	19	0	1	0	0	10	5	6	13	5				
17	grey	1	1	0	1	0	0	3	13	10	0	1				
18	lightblue	0	1	0	0	0	0	9	17	22	1	16				
19	grey	2	2	0	1	0	0	15	9	10	5	2				
20	lightblue	0	1	1	0	0	0	0	23	18	0	26				
21	grey	3	12	1	0	0	0	10	12	5	6	6				
22	grey	0	11	0	0	0	0	15	13	4	9	4				
23	grey	0	13	1	0	0	0	17	4	4	12	2				
24	grey	0	15	1	0	0	0	13	7	4	4	3				
25	gold	0	5	1	0	0	0	13	12	5	23	2				
26	lightblue	0	2	0	1	0	0	19	9	10	9	4				
27	lightblue	0	1	0	1	0	1	1	16	18	0	58				
28	grey	0	5	1	0	1	0	10	8	7	11	5				
29	lightblue	0	0	0	3	0	0	1	16	23	0	20				
30	lightblue	5	1	0	1	0	0	0	13	14	1	53				
31	lightblue	1	1	0	4	0	1	0	9	16	0	33				
		91	260	60	136	17	28	315	296	286	180	330	1999			

Supplementary Table 2: GSEA report of rank differentially expressed genes of cluster 1 cells.

NAME	GS<br>follow link to MSigDB	GS DETAILS	SIZE	ES	NES	NOM p-val	FDR q-val	FWER p-val	RANK AT MAX	LEADING EDGE	
HALLMARK	HALLMARK_INTERFERON_GAMMA_RESPONSE	Details ...	96	0.835332	2.970258	0	0	0	210	tags=61%, list=7%, signal=64%	
HALLMARK	HALLMARK_INTERFERON_ALPHA_RESPONSE	Details ...	58	0.871693	2.816744	0	0	0	210	tags=71%, list=7%, signal=75%	
HALLMARK	HALLMARK_INFLAMMATORY_RESPONSE	Details ...	38	0.761998	2.293237	0	0	0	232	tags=39%, list=8%, signal=42%	
HALLMARK	HALLMARK_ALLOGRAFT_REJECTION	Details ...	67	0.547623	1.82981	0	0.015362	0.052	184	tags=27%, list=6%, signal=28%	
HALLMARK	HALLMARK_IL2_STAT5_SIGNALING	Details ...	47	0.556718	1.745757	0.007246	0.026888	0.109	179	tags=23%, list=6%, signal=25%	
HALLMARK	HALLMARK_HYPOXIA	Details ...	40	0.532786	1.618245	0.019868	0.068344	0.302	425	tags=38%, list=15%, signal=43%	
HALLMARK	HALLMARK_FATTY_ACID_METABOLISM	Details ...	50	0.500853	1.602942	0.021368	0.065523	0.333	275	tags=22%, list=9%, signal=24%	
HALLMARK	HALLMARK_TNFA_SIGNALING_VIA_NFKB	Details ...	40	0.532225	1.588731	0.037862	0.062829	0.358	444	tags=43%, list=15%, signal=49%	
HALLMARK	HALLMARK_APOPTOSIS	Details ...	51	0.486255	1.556311	0.02331	0.069677	0.426	241	tags=24%, list=8%, signal=25%	
HALLMARK	HALLMARK_IL6_JAK_STAT3_SIGNALING	Details ...	29	0.478313	1.370885	0.111607	0.195153	0.812	426	tags=48%, list=15%, signal=56%	
HALLMARK	HALLMARK_GLYCOLYSIS	Details ...	47	0.396216	1.237715	0.2	0.35539	0.971	651	tags=38%, list=22%, signal=48%	
HALLMARK	HALLMARK_UV_RESPONSE_UP	Details ...	50	0.366291	1.161108	0.260369	0.458239	0.997	303	tags=18%, list=10%, signal=20%	
HALLMARK	HALLMARK_KRAS_SIGNALING_UP	Details ...	39	0.37853	1.149584	0.2625	0.443623	0.997	9	tags=5%, list=0%, signal=5%	
HALLMARK	HALLMARK_P53_PATHWAY	Details ...	46	0.339248	1.071395	0.346756	0.553029	0.999	302	tags=24%, list=10%, signal=26%	
HALLMARK	HALLMARK_EPITHELIAL_MESENCHYMAL_TRANSITION	Details ...	20	0.395773	1.060681	0.384783	0.536904	0.999	426	tags=35%, list=15%, signal=41%	
HALLMARK	HALLMARK_CHOLESTEROL_HOMEOSTASIS	Details ...	15	0.355497	0.875187	0.600467	0.896738	1	628	tags=53%, list=21%, signal=68%	
HALLMARK	HALLMARK_MTORC1_SIGNALING	Details ...	105	0.234575	0.854284	0.765517	0.887126	1	501	tags=26%, list=17%, signal=30%	
HALLMARK	HALLMARK_MYC_TARGETS_V1	Details ...	156	0.220086	0.847931	0.805936	0.850472	1	517	tags=24%, list=18%, signal=28%	
HALLMARK	HALLMARK_ANDROGEN_RESPONSE	Details ...	30	0.289823	0.829187	0.690531	0.838284	1	8	tags=3%, list=0%, signal=3%	
HALLMARK	HALLMARK_MYC_TARGETS_V2	Details ...	29	0.279024	0.821365	0.717195	0.811555	1	285	tags=17%, list=10%, signal=19%	
HALLMARK	HALLMARK_OXIDATIVE_PHOSPHORYLATION		126	0.199282	0.742041	0.945148	0.891897	1	766	tags=37%, list=26%, signal=47%	
HALLMARK	HALLMARK_DNA_REPAIR		78	0.17807	0.617595	0.984946	0.959471	1	290	tags=8%, list=10%, signal=8%	
Red=significant											

Supplementary Table 3: information on human samples

Sample ID	Blasts' Immunophenotype	Molecular
160005	from Rambam: 24% blasts at BM all: CD117+/CD13+/CD38+/CD11b-/CD16/CD15-/CD64-/CD33+/CD10-/DR+/CD123-/CD34-/MPO+ Could fit AML M1/M2 . cyCD19=0% ,cyCD3=0% ,CD64=0% ,CD123=0%	*DNMT3A* R882, NPM1c
141464		*DNMT3A* R882
141164		*DNMT3A* R882, 3 different subclones TET2 M1333K, ASXL1, JAK2 V617F
141519		WT
141467		*DNMT3A* R882
150279	Positive for CD117 (dim equivocal), CD11b (predominantly negative, subset dim),CD33, CD38 (dim), CD71 (equivocal), CD123, CD11c, CD64 (very dim), with myeloperoxidase. Negative for CD13, CD14, CD15, CD16, CD36, CD56, CD65, HLA-DR, CD235a, CD19, CD10, cCD22, cCD79a, cCD3,CD2, CD4, CD7, or nTdT	FLT3-ITD+, FLT3-TKD-, NPM1+

Supplementary Table 4: Antibodies and viability staining used for flow cytometry

Antibody.	Manufacturer	Clone/Catalogue num.	Titer
Anti mouse CD45.2-APC	BioLegend, San Diego, CA, USA	104/109814	1:200
Anti mouse CD45.1-PE	BioLegend, San Diego, CA, USA	A20/110708	1:200
Rat anti mouse CD4 -FITC	BioLegend, San Diego, CA, USA	GK1.5/100408	1:200
Rat anti mouse B220-FITC	BioLegend, San Diego, CA, USA	RA3-6B2/103208	1:200
Rat anti mouse Gr1-FITC	BioLegend, San Diego, CA, USA	RB6-8C5/108406	1:200
Rat anti mouse CD11b-FITC	BioLegend, San Diego, CA, USA	M1/70/101206	1:200
Rat anti mouse CD8a-FITC	BioLegend, San Diego, CA, USA	53-6.7 EF450/100706	1:200
Rat anti mouse Ter119-FITC	BioLegend, San Diego, CA, USA	TER-119/116206	1:200
Rat anti mouse c-kit- BV605	BioLegend, San Diego, CA, USA	2B8/105847	1:500
Rat anti mouse Sca-1-PE-Vio770	Miltenyi Biotec	D7/130-102-832	1:500
Mouse anti humanCD45-BV510	BioLegend, San Diego, CA, USA	HI30/304036	1:200
Mouse anti human CD33-APC	BD Biosciences, San Jose, CA, USA	WM53/561817	1:100
Mouse anti human CD34-APC Cy7	BioLegend, San Diego, CA, USA	581/343514	1:100
Mouse anti human CD15-BV421	BioLegend, San Diego, CA, USA	W6D3/323040	1:100
Mouse anti human CD38-PE Cy7	BioLegend, San Diego, CA, USA	HIT2/980312	1:100
Mouse anti human CD3-FITC	BD Biosciences, San Jose, CA, USA	UCHT1/561806	1:100
Mouse anti human CD19-PE	BD Biosciences, San Jose, CA, USA	HIB19/555413	1:200

References

1. Baran, Y. *et al.* MetaCell: analysis of single-cell RNA-seq data using K-nn graph partitions. *Genome Biol* **20**, (2019).
2. Ben-Kiki, O., Bercovich, A., Lifshitz, A. & Tanay, A. Metacell-2: a divide-and-conquer metacell algorithm for scalable scRNA-seq analysis. *Genome Biol* **23**, 100 (2022).
3. Baccin, C. *et al.* Combined single-cell and spatial transcriptomics reveal the molecular, cellular and spatial bone marrow niche organization. *Nat Cell Biol* **22**, 38–48 (2020).
