# Supplementary material for: Inflammatory signals from fatty bone marrow support DNMT3A driven clonal hematopoiesis
Source: Nat Commun. 2023 Apr 12;14:2070. doi: 10.1038/s41467-023-36906-1 (PMC10097668; doi:10.1038/s41467-023-36906-1)
Supplement: Supplementary file 3 — Reporting Summary [file 41467_2023_36906_MOESM3_ESM.pdf]

nature portfolio

Corresponding author(s): Dr. Liran Shlush

Last updated by author(s): 2023-1-23

Reporting Summary

Nature Portfolio wishes to improve the reproducibility of the work that we publish. This form provides structure for consistency and transparency in reporting. For further information on Nature Portfolio policies, see our Editorial Policies and the Editorial Policy Checklist.

Statistics

For all statistical analyses, confirm that the following items are present in the figure legend, table legend, main text, or Methods section.

n/a	Confirmed	
☐	☒	The exact sample size (*n*) for each experimental group/condition, given as a discrete number and unit of measurement
☐	☒	A statement on whether measurements were taken from distinct samples or whether the same sample was measured repeatedly
☐	☒	The statistical test(s) used AND whether they are one- or two-sided<br>*Only common tests should be described solely by name; describe more complex techniques in the Methods section.*
☐	☒	A description of all covariates tested
☐	☒	A description of any assumptions or corrections, such as tests of normality and adjustment for multiple comparisons
☐	☒	A full description of the statistical parameters including central tendency (e.g. means) or other basic estimates (e.g. regression coefficient) AND variation (e.g. standard deviation) or associated estimates of uncertainty (e.g. confidence intervals)
☐	☒	For null hypothesis testing, the test statistic (e.g. *F*, *t*, *r*) with confidence intervals, effect sizes, degrees of freedom and *P* value noted<br>*Give P values as exact values whenever suitable.*
☒	☐	For Bayesian analysis, information on the choice of priors and Markov chain Monte Carlo settings
☒	☐	For hierarchical and complex designs, identification of the appropriate level for tests and full reporting of outcomes
☒	☐	Estimates of effect sizes (e.g. Cohen's *d*, Pearson's *r*), indicating how they were calculated

Our web collection on statistics for biologists contains articles on many of the points above.

Software and code

Policy information about availability of computer code

Data collection	No software was used for data collection.
Data analysis	All flow cytometry analyses were performed on FirePlex (Beckman Coulter, Brea, CA, USA), using CytExpert software v 2.4.0.28 (Beckman Coulter, Brea, CA, USA). image stream:data were analyzed using the manufacturer's image analysis software (IDEAS 6.2; Amnis).

For manuscripts utilizing custom algorithms or software that are central to the research but not yet described in published literature, software must be made available to editors and reviewers. We strongly encourage code deposition in a community repository (e.g. GitHub). See the Nature Portfolio guidelines for submitting code & software for further information.

Data

Policy information about availability of data

All manuscripts must include a data availability statement. This statement should provide the following information, where applicable:

- Accession codes, unique identifiers, or web links for publicly available datasets
- A description of any restrictions on data availability
- For clinical datasets or third party data, please ensure that the statement adheres to our policy

The sequencing data and processed data generated in this study have been deposited in the ArrayExpress database (https://www.ebi.ac.uk/biostudies/arrayexpress) under accession codes: E-MTAB-12613, E-MTAB-12614, E-MTAB-12620

Human research participants

Policy information about studies involving human research participants and Sex and Gender in Research.

Reporting on sex and gender	Not applicable
Population characteristics	This study involved primary samples derived from 6 individuals. Clinical characteristics are reported in supplementary table 3.
Recruitment	All primary samples were preexisting and obtained from Leukemia Tissue Bank Princess Margaret Cancer Center or Rambam Health Care Campus. We do not know of any selection biases during recruitment of these samples.
Ethics oversight	All human samples were collected, Ficoll separated and viably frozen with informed consent according to procedures approved by Rambam Health Care Campus, Haifa, Israel IRB # 0280-09-RMB and the university health network (UHN) IRB # 01-0573. All samples were sequenced with our in house clonal hematopoiesis panel 65. Information on all samples can be found in the manuscript

Note that full information on the approval of the study protocol must also be provided in the manuscript.

Field-specific reporting

Please select the one below that is the best fit for your research. If you are not sure, read the appropriate sections before making your selection.

☒ Life sciences ☐ Behavioural & social sciences ☐ Ecological, evolutionary & environmental sciences

For a reference copy of the document with all sections, see nature.com/documents/nr-reporting-summary-flat.pdf

Life sciences study design

All studies must disclose on these points even when the disclosure is negative.

Sample size	All relevant sample sizes are described in the legends in the figure and/or the methods section. Sample sizes were determined based on prior experiences with each of the experimental systems and their expected variability. No statistical testing was used to predetermine sample size
Data exclusions	No data were excluded from the analysis.
Replication	Three or more biological experiments. The results were successfully replicated.
Randomization	Mice were randomly assigned to different groups and samples used in vitro experiments were randomly assigned to each group.
Blinding	No blinding was used in our experiments, since the readouts were quantitative and not prone to subjective judgment of investigators.

Reporting for specific materials, systems and methods

We require information from authors about some types of materials, experimental systems and methods used in many studies. Here, indicate whether each material, system or method listed is relevant to your study. If you are not sure if a list item applies to your research, read the appropriate section before selecting a response.

Materials & experimental systems

n/a	Involved in the study	
☐	☒	Antibodies
☒	☐	Eukaryotic cell lines
☒	☐	Palaeontology and archaeology
☐	☒	Animals and other organisms
☒	☐	Clinical data
☒	☐	Dual use research of concern

Methods

n/a	Involved in the study	
☒	☐	ChIP-seq
☐	☒	Flow cytometry
☒	☐	MRI-based neuroimaging

Antibodies

Antibodies used	Anti mouse CD45.2-APC BioLegend, San Diego, CA, USA 104/109814 1:200<br>Anti mouse CD45.1-PE BioLegend, San Diego, CA, USA A20/110708 1:200<br>Rat anti mouse CD4 -FITC BioLegend, San Diego, CA, USA GK1.5/100408 1:200

Rat anti mouse B220-FITC BioLegend, San Diego, CA, USA RA3-6B2/103208 1:200
Rat anti mouse Gr1-FITC BioLegend, San Diego, CA, USA RB6-8C5/108406 1:200
Rat anti mouse CD11b-FITC BioLegend, San Diego, CA, USA M1/70/101206 1:200
Rat anti mouse CD8a-FITC BioLegend, San Diego, CA, USA 53-6.7 EF450/100706 1:200
Rat anti mouse Ter119-FITC BioLegend, San Diego, CA, USA TER-119/116206 1:200
Rat anti mouse c-kit- BV605 BioLegend, San Diego, CA, USA 2B8/105847 1:500
Rat anti mouse Sca-1-PE-Vio770 Miltenyi Biotec D7/130-102-832 1:500
Mouse anti humanCD45-BV510 BioLegend, San Diego, CA, USA HI30/304036 1:200
Mouse anti human CD33-APC BD Biosciences, San Jose, CA, USA WM53/561817 1:100
Mouse anti human CD34-APC Cy7 BioLegend, San Diego, CA, USA 581/343514 1:100
Mouse anti human CD15-BV421 BioLegend, San Diego, CA, USA W6D3/323040 1:100
Mouse anti human CD38-PE Cy7 BioLegend, San Diego, CA, USA HIT2/980312 1:100
Mouse anti human CD3-FITC BD Biosciences, San Jose, CA, USA UCHT1/561806 1:100
Mouse anti human CD19-PE BD Biosciences, San Jose, CA, USA HIB19/555413 1:200

Validation

All antibodies were commercially validated for use in flow cytometry on mouse and human cells by manufactures: BioLegend, Milltenyi and BD Bioscience. Full validation statements available on manufactures websites.

Animals and other research organisms

Policy information about studies involving animals; ARRIVE guidelines recommended for reporting animal research, and Sex and Gender in Research

Laboratory animals

8-12 weeks male immune-deficient NSG (NOD/SCID/IL-2Rgc-null) mice: NSG (Stock No: 005557) (The Jackson Laboratory, Bar-Harbor, ME, USA). NOD.Cg-Prkdcscid Il2rgtm1Wjl Tg(PGK1-KITLG*220)441Daw/SzJ mice (stock No: 017830) (The Jackson Laboratory, Bar-Harbor, ME, USA). NOD.Cg-Prkdcscid Il2rgtm1Wjl Tg(CMV-IL3,CSF2,KITLG)1Eav/MloySzJ mice (Stock No: 013062) NSG-SGM3(The Jackson Laboratory, Bar-Harbor, ME, USA) DNMT3AR882H KI mice, constitutively express the human DNMT3A mutation. SRSF2P95H floxed mice possess loxP sites flanking the endogenous coding region of the serine/arginine-rich splicing factor 2 (SRSF2) gene (Stock No: 028376) (The Jackson Laboratory, Bar-Harbor, ME, USA). DNMT3AR882H or SRSF2P95H were crossed with VAV Cre (Stock No: 008610) (The Jackson Laboratory, Bar-Harbor, ME, USA). Mice were kept 8-12 weeks male immune-deficient NSG (NOD/SCID/IL-2Rgc-null) mice: NSG (Stock No: 005557) (The Jackson Laboratory, Bar-Harbor, ME, USA). NOD.Cg-Prkdcscid Il2rgtm1Wjl Tg(PGK1-KITLG*220)441Daw/SzJ mice (stock No: 017830) (The Jackson Laboratory, Bar-Harbor, ME, USA). NOD.Cg-Prkdcscid Il2rgtm1Wjl Tg (CMV-IL3,CSF2,KITLG)1Eav/MloySzJ mice (Stock No: 013062) NSG-SGM3(The Jackson Laboratory, Bar-Harbor, ME, USA) DNMT3AR882H KI mice, constitutively express the human DNMT3A mutation. SRSF2P95H floxed mice possess loxP sites flanking the endogenous coding region of the serine/arginine-rich splicing factor 2 (SRSF2) gene (Stock No: 028376) (The Jackson Laboratory, Bar-Harbor, ME, USA). DNMT3AR882H or SRSF2P95H were crossed with VAV Cre (Stock No: 008610) (The Jackson Laboratory, Bar-Harbor, ME, USA). Mice were kept in accordance with the law and institutional regulations. All mice were maintained under 12 hours dark/light cycle, at an ambient temperature of around 22 degrees and humidity of 50%.

Wild animals

The study did not involve wild animals

Reporting on sex

In this study, male castration used as one of our fatty bone marrow models. Considering that the castration model recapitulates the age-related decline in male testosterone. As a result, male mice were used in this study for all the experiments.

Field-collected samples

No field collected samples were used in this study

Ethics oversight

All experiments were performed in accordance with institutional guidelines approved by the Weizmann Institute of Science Animal Care Committee (11790319-2).

Note that full information on the approval of the study protocol must also be provided in the manuscript.

Flow Cytometry

Plots

Confirm that:

- [x] The axis labels state the marker and fluorochrome used (e.g. CD4-FITC).
- [x] The axis scales are clearly visible. Include numbers along axes only for bottom left plot of group (a 'group' is an analysis of identical markers).
- [x] All plots are contour plots with outliers or pseudocolor plots.
- [x] A numerical value for number of cells or percentage (with statistics) is provided.

Methodology

Sample preparation

Freshly dissected femora and tibiae were isolated from two months old or one-year mice DNMT3AMut, DNMT3A haplo, DNMT3AWT, SRSF2Mut or control SRSF2WT mice CD45.2. BM was flushed with a 1cc (23G) into IMDM (Iscove's Modified

Dulbecco's Medium). The BM was spun at 0.3 g by centrifugation and RBCs were lysed in ammonium chloride-potassium bicarbonate lysis buffer for 1 min. After centrifugation, cells were resuspended in PBS, passed through a cell strainer, and counted. Then 6x10^6 cells were injected intra femur into NSG (CD45.1) mice that were Irradiated (FBM) seven days before with low dose Irradiation (225 rad) or to non-Irradiated (CONTROL) NSG mice. Eight weeks following cells transfer, mice were sacrificed. Right femur and the other bones (left femur and tibias) were cut and BM cells were flushed with IMDM (Iscove's Modified Dulbecco's Medium) and analyzed by FACS

Instrument	All flow cytometry analyses were performed on FirePlex (Beckman Coulter, Brea, CA, USA), using CytExpert software v 2.4.0.28
Software	CytExpert software v 2.4.0.28
Cell population abundance	Engraftment was assessed according to presence of ≥0.1% mCD45.2 cells.<br>LSK represented about 2% of total fatty Bone Marrow population cells (Supplementary Figure 14b)
Gating strategy	Multilineage engraftment of AML patient-derived analysis : Cells were gated by using forward scatter height (FSC-H) vs. Forward scatter area (FSC-A) plots and side scatter height (SSC-H) vs. side scatter area (SSC-A) to exclude doublets. Live cells were gated as negative to propidium iodide staining. Then cells were gated on CD45 expression. A multi-lineage engraftment was defined when a subpopulation of B cell progenitors CD33-CD3- cells expressing CD19+.<br><br>LSK analysis: Cells were gated by using forward scatter height (FSC-H) vs. Forward scatter area (FSC-A) and FSC-w vs. FSC-A to exclude doublets. To further analyze LSK, Cells were gated on CD45.2 cell population from which Lin negative cells were gated. Then cKit and Sca1 expression was analyzed on Lin- cells (Supplementary Figure 14a).

☒ Tick this box to confirm that a figure exemplifying the gating strategy is provided in the Supplementary Information.
