# Supplementary material for: Inflammatory signals from fatty bone marrow support DNMT3A driven clonal hematopoiesis
Source: Nat Commun. 2023 Apr 12;14:2070. doi: 10.1038/s41467-023-36906-1 (PMC10097668; doi:10.1038/s41467-023-36906-1)
Supplement: Supplementary file 4 — Description of Additional Supplementary Files [file 41467_2023_36906_MOESM4_ESM.pdf]

Description of Additional Supplementary Files

Supplementary Data 1: Expression between the different clusters in the UMAP

Supplementary Data 2: Data for Fig 4

Supplementary Data 3: Single RNA scores

Supplementary Data 4: Single RNA scores
